# Illuminating the Invisible: Green Fluorescent Protein as a Beacon for Antibiotic-Induced Phage Activity in *Escherichia coli*

**DOI:** 10.3390/antibiotics14070714

**Published:** 2025-07-16

**Authors:** Maria João Silva, Tim Van Den Bossche, Mattias Collin, Rolf Lood

**Affiliations:** 1 Division of Infection Medicine, Department of Clinical Sciences, Faculty of Medicine, Lund University, SE-22184 Lund, Sweden; mattias.collin@med.lu.se; 2 VIB-UGent Center for Medical Biotechnology, VIB, 9052 Ghent, Belgium; tim.vandenbossche@ugent.be; 3 Department of Biomolecular Medicine, Faculty of Medicine and Health Sciences, Ghent University, 9052 Ghent, Belgium

**Keywords:** bacteriophages, antibiotic resistance, transduction, fluorescence, ddPCR

## Abstract

**Background/Objectives:** Antibiotic resistance presents an urgent public health threat. By developing a streamlined and effective method for studying bacteriophage induction, this research marks a step further in understanding how antibiotic-resistant genes might spread across different environments. This knowledge is essential for creating strategies to reduce the spread of antimicrobial resistance (AMR), particularly from a One Health perspective. In this study, we develop and validate a Green Fluorescent Protein (GFP)-based method as a proxy for bacteriophage induction. This method screens compounds for their potential to promote bacteriophage induction. **Methods:** This study utilized a *recA*-*GFP* construct in *Escherichia coli* to measure fluorescence as an indicator of SOS response activation. The experiments involved treating *E. coli* cultures with varying concentrations of the DNA-damaging chemical mitomycin C and measuring fluorescence over time. Additionally, droplet digital PCR (ddPCR) quantified bacteriophage induction in a lambda phage-carrying *E. coli* strain, allowing for correlation analysis between the two methods. **Results:** The *recA*-driven SOS response depended on both dose and time, with increasing concentrations of mitomycin C leading to higher fluorescence. ddPCR analysis confirmed that mitomycin C induced prophage activation, with gene ratios increasing at higher drug concentrations over time. A strong Spearman correlation (>0.7) was noted between fluorescence and ddPCR results at elevated concentrations and relevant time points, indicating the validity of the GFP-based model as a proxy for bacteriophage induction. **Conclusions:** The findings demonstrate a strong association between the two methods of measuring phage induction, suggesting that the GFP-based *E. coli* model is a reliable, cost-effective, and efficient tool for studying phage induction and its potential role in AMR spread. This method could facilitate the screening of environmental samples and specific drugs to evaluate their impact on bacteriophage induction, which opens the door for applications such as screening for antibiotic resistance dissemination.

## 1. Introduction

Antibiotic resistance, in particular, the inability of antibiotics to act on bacteria, occurs when changes in bacteria cause antibiotics to be less effective against them upon infection [1]. Although this topic is constantly discussed, the WHO considers it one of this century’s leading public health threats and believes that it needs to be addressed urgently [2]. It seems even more concerning when viewed from a One Health perspective.

One Health is a concept that integrates the health of humans, animals, plants, and ecosystems as a whole and states that for the human population to be healthy, the ecosystem around it also needs to be healthy, so when assessing one group, all the others need to be taken into consideration due to them coexisting and being interdependent [3]. The association between humans and animals, and consequently the ecosystems where they live, cannot be ignored. Evidence shows that the inadequate use of antibiotics in human and animal health has contaminated their surrounding environments. Environmental reservoirs of antibiotic resistance play a role in its dissemination [4,5]. These reservoirs encompass soil, water, hospital, industrial, and farm waste, and wastewater treatment plants [4,5,6].

Phages are the most abundant gene transfer vehicles. They can infect bacteria, cause their lysis, stay dormant, and even alter bacterial pathogenicity, conferring on them virulence factor gene-carrying prophages [6,7,8,9]. Upon infection, phages can display one of two principal life cycles: lytic or lysogenic. In the lytic cycle, phages multiply inside the host cell before releasing new phage particles [10]. In the lysogenic cycle, phages infect bacteria, incorporate their genome into the host genome, and replicate as part of the host genome as prophages [8,10]. By integrating into their host’s genome, they can also uptake parts of it and transfer it to other bacteria. This mechanism, called transduction, is considered one of the major mechanisms for horizontal gene transfer and a primary driving force of evolution [6,9,10]. Prophages can eventually switch to a lytic cycle upon cues that usually involve host stress [7].

Bacteria are continuously exposed to stressful and changing environments, which leads them to develop response strategies and mechanisms, one of the most significant being the SOS response [11]. The SOS response is an inducible DNA repair pathway that promotes cell survival upon interrupted replication and DNA damage [12]. This mechanism is primarily controlled by two key proteins, LexA (a transcriptional repressor) and RecA (a recombinase that acts like an inducer). Upon DNA damage, RecA binds to ssDNA (activation and conversion of RecA into RecA*), leading to the self-cleavage of LexA and the activation of more than 50 SOS genes in *Escherichia coli*, which ultimately culminate with DNA repair [11,13]. The SOS response mechanism can be triggered by both endogenous and exogenous stimuli, UV and gamma radiation, antibiotics and other drugs, oxidants, chemical mutagens, and even our own bodies [11,13,14].

The SOS response mechanism is activated not only during DNA damage and antibiotic induction but also during horizontal gene transfer events, such as conjugation, transformation, or the uptake of exogenous DNA in the form of plasmids, since these events can lead to the formation of ssDNA, which induces the SOS response [13]. This widely conserved and widespread mechanism is extremely important when discussing antibiotic resistance. Some antibiotics cause the formation of ssDNA, which induces the SOS response [13]. Recent studies have shown that sub-MIC (Minimum Inhibitory Concentration) levels of certain SOS-activating agents, such as mitomycin C, induce resistance and increase tolerance to such drugs [12,13,15,16]. Thus, this response represents a target for improving therapeutic protocols.

Mitomycin C is a naturally occurring antibiotic and antitumor drug discovered during the 1950s, widely used in the clinic in chemotherapy due to its ability to disrupt DNA replication [12,17]. This ability also induces the SOS response in bacteria, making mitomycin C an important drug for studying this mechanism.

Recent studies suggest that up to 75% of published bacterial genomes contain prophage sequences, with some strains of *E. coli*, *Lactococcus lactis*, and *Streptococcus pyogenes* having up to 8% of their genome consisting of prophage sequences [18,19]. Upon SOS response activation in a bacterium carrying a prophage, the prophage will be induced and replicated and DNA will be packaged into its capsid—DNA that can encode resistance genes—that can later spread to other bacteria through transduction [6]. It is thus important to understand what dictates such phage activation and, more specifically, what substances trigger phage induction.

In this study, we developed and validated a GFP-based *E. coli* model as a proxy for bacteriophage induction. This model can be used to screen any compounds or environments that promote bacteriophage induction, opening the door to applications such as screening for antimicrobial resistance. We hypothesize that the model we developed is a reliable tool, and its results can be correlated with those obtained through ddPCR.

## 2. Results

### 2.1. MIC Measurements Are Similar for recA-GFP and Wild-Type K12 Strains

To ensure that our assay would be performed with sub-MIC concentrations of mitomycin C, MIC values needed to be tested for the recA-GFP strain used (MG1655 K12). The results indicate that the mitomycin C MIC values for the aforementioned strain are the same as those for the K12 wild-type strain, approximately 8 μg/mL.

### 2.2. recA-Induction by Mitomycin C Is Dose- and Time-Dependent

**Figure 1:**
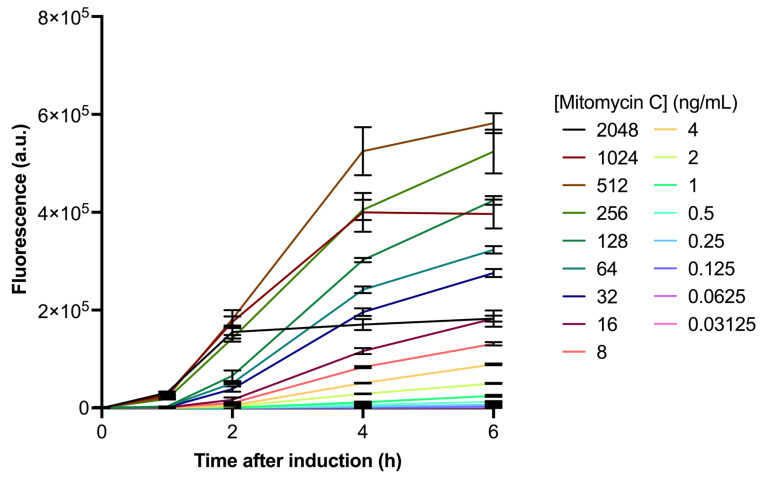
Fluorescence intensity (in arbitrary units) over time after induction (in hours) with varying concentrations of mitomycin C (ranging from 2048 ng/mL to 0.03125 ng/mL), showing the relationship between concentration and fluorescence response.

To evaluate whether the *recA-*driven SOS response could serve as a proxy for bacteriophage induction, we examined its reaction to the commonly used prophage-inducing drug mitomycin C across various time points and doses. Mitomycin C efficiently triggers fluorescence intensity, which increases over time after induction. Higher concentrations of mitomycin C result in greater fluorescence (Figure 1). It can also be noted that at 2048 ng/mL and 1024 ng/mL of mitomycin C, the fluorescence reaches a plateau, while at 512 ng/mL and below, the fluorescence values directly correlate with the drug dosage, with higher doses emitting stronger fluorescence and lower doses emitting less fluorescence.

### 2.3. Mitomycin C Induction of Bacteriophage Can Efficiently Be Quantified by ddPCR

**Figure 2:**
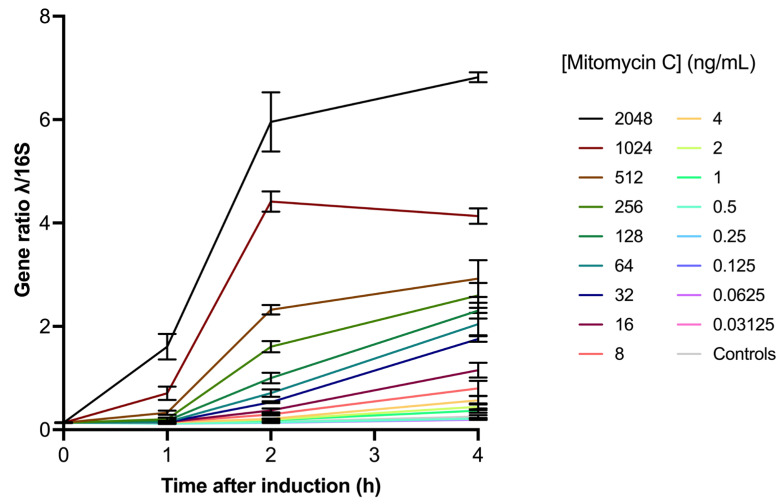
Relative abundance of ddPCR-amplified λ genes to 16S genes over time after induction (in hours), with mitomycin C doses varying in concentrations from 2048 to 0.03125 ng/mL.

To verify that not only was *recA* activated by mitomycin C, but that actual prophage induction was initiated, we repeated the experimental setup in a lambda (λ) phage-carrying *E. coli* strain, analyzing phage induction using droplet digital polymerase chain reaction (ddPCR). Similarly, we detected increases in the gene ratio (lambda/16S) over time. The gene ratio is also higher with increased mitomycin C doses and decreases with lower drug concentrations (Figure 2).

### 2.4. Phage Induction Correlates Well with recA Activation

**Figure 3:**
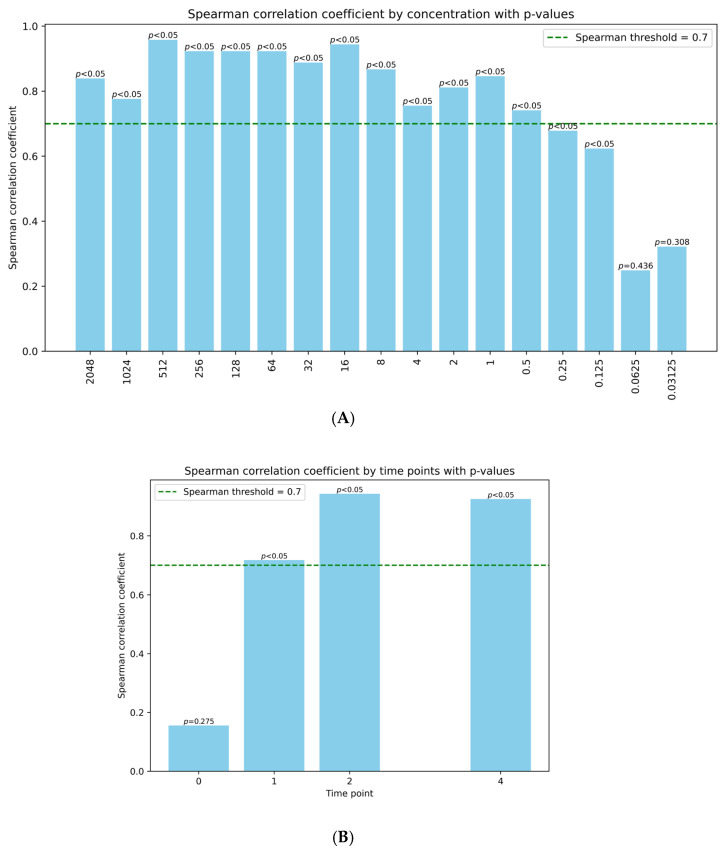
(**A**) Spearman correlation coefficients by concentration of mitomycin C (in ng/mL). A threshold was set at 0.7 (dotted line), and *p*-values are shown above each bar. (**B**) Spearman correlation coefficients by time after induction time points (in hours). A threshold was set at 0.7 (dotted line), and *p*-values are shown above each bar.

**Figure A1:**
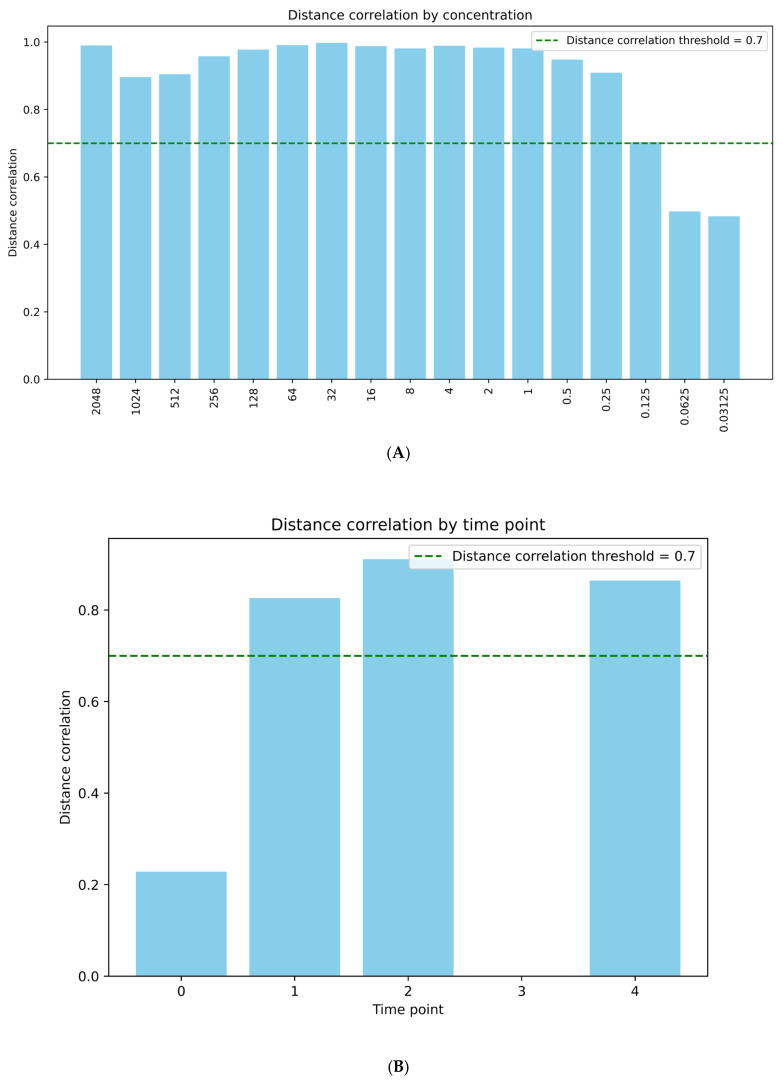
Distance correlations. (**A**) Illustration of the distance correlation coefficients across various mitomycin C concentrations, ranging from 2048 ng/mL to 0.03125 ng/mL, for phage lambda induction and GFP. (**B**) Distance correlation coefficients for different time points between phage lambda induction and GFP.

To validate the use of *recA* induction as a model for studying bacteriophage induction, we investigated whether the outcomes of the two different methods correlated. Figure 3A illustrates the Spearman correlation coefficients between the various mitomycin C concentrations in both methods and their corresponding *p*-values. The data points are plotted to show the relationship between concentration levels and the strength of correlation. We set a threshold at 0.7; values above this threshold indicate a strong positive correlation. Mitomycin C concentrations between 0.125 and 2048 ng/mL showed a significant correlation with phage induction, with 0.5–2048 ng/mL also being above the threshold of 0.7. Similarly, evaluating the impact of time on the correlation between the GFP signal and actual phage induction in our model demonstrated a good correlation at all time points (Figure 3B). Similar results were achieved using a distance correlation model (Figure A1).

## 3. Discussion

Phage induction is a crucial mechanism for spreading antibiotic resistance and virulence factors, making it an important study area. However, our understanding of what substances (such as antibiotics or chemicals) trigger this phenomenon remains limited, partly due to the currently employed cumbersome and resource-intensive (expensive or time-consuming) methods. With this new validated method, it will be possible to screen environmental samples, specific targeted drugs, and other potential stressors to evaluate their impact on antibiotic spread.

In our developed method, we demonstrated that phage induction can be estimated through the induction of the SOS response in a high-throughput assay, with significant correlative values across a wide range of stimuli concentrations as well as time points. It should be noted that the phage/16S ratio is expected to be 1:1 at the initiation of the experiment since the phage lambda exists as one copy integrated within the genome of *E. coli*. However, likely due to inefficient PCR setups, the initial ratio is significantly lower. Nonetheless, since the purpose of the assay was not to demonstrate *absolute* changes but to demonstrate *relative* changes and correlate such changes with GFP, the achieved results are adequate, and we did not, therefore, optimize this step further.

However, limitations must also be considered. The phage induction measured through fluorescence serves as a proxy rather than a verified true phage induction, and the actual response may vary between different strains and species of bacteria. Furthermore, despite *recA*-driven induction of phages being one of the most potent phage-inducing mechanisms, other mechanisms occur that will not be captured with this assay. Still, for screening purposes and in combination with ddPCR for a subset of samples, it has great potential. Additionally, not all *recA* activation will result in phage activation. Nevertheless, the herein-developed tool may prove to be a beneficial method to rapidly screen substances (e.g., antibacterial drugs, environmental samples, water) to generate a map of potential phage-inducing substances and, therefore, AMR-driving substances. In the future, it would be interesting to follow up on this method with whole-genome sequencing to identify phage genetic material and transferred genes, which would provide a deeper understanding of the underlying resistance mechanisms.

## 4. Materials and Methods

### 4.1. MIC Measurements

The MIC was measured according to “Protocol 2a. MIC determination using broth microdilution”, described by Kadeřábková et al. [20], with the following alterations: (1) cultures were grown directly from frozen in liquid medium, and (2) a sterile saline solution of 0.9% *w*/*v* was used instead of 0.85% *w*/*v*. The mitomycin C concentrations tested ranged from 32,768 ng/mL to 512 ng/mL.

### 4.2. recA-GFP Induction in E. coli Cells

Overnight cultures of *E. coli* MG1655 K12 carrying the *recA*-GFP construct pUA66: gfpmut2 [21] (37 °C, 175 rpm, 50 μg/mL kanamycin) were subcultured in fresh LB (1:20) until they reached the early log phase (OD_600_ = 0.3). The bacterial culture was diluted until OD_600_ = 0.1 and added to a black 96-well plate together with a blank (PBS) or positive control (mitomycin C, 0.03125–2048 ng/mL) in a 1:1 ratio (*v*:*v*). Fluorescence (485/535 nm) was measured at 0–6 h. All samples were analyzed in experimental (*n* = 3) and technical (*n* = 4) replicates.

### 4.3. ddPCR Analysis of Bacteriophage Induction in E. coli

Overnight *E. coli* cultures carrying a λ phage K12 LE392 (DSMZ, Leibniz, Germany) (37 °C, 175 rpm) were subcultured in fresh LB (1:20) until they reached the early log phase (OD_600_ = 0.3). The bacterial culture was diluted until OD_600_ = 0.1 and added to 50 mL tubes together with a blank (PBS) or positive control (mitomycin C, 0.03125–2048 ng/mL) in a 1:1 ratio (*v*:*v*).

Samples were collected and frozen at 0, 1, 2, and 4 h after induction. In between measurements, cultures were kept at 37 °C.

DNA was extracted by boiling the frozen samples at 105 °C for 5 min and centrifuging at 14,000× *g* for 10 min. The lysate containing DNA was isolated and used for the ddPCR measurements. Absolute quantification of gene copies was conducted on a *Bio-Rad QX200 Droplet Digital system* (Bio-Rad, Hercules, CA, USA) according to the manufacturer’s instructions, using primers specific to 16S *E. coli* and for the lambda phage (Table 1). The PCR was conducted in a *Bio-Rad C1000 thermal cycler* (Bio-Rad, Hercules, CA, USA), following standard ddPCR cycling settings as recommended by the manufacturer. Amplified products were analyzed in a *QX200 Droplet Reader* (Bio-Rad, Hercules, CA, USA), and the data was analyzed with *QX Manager Software 2.2 Standard Edition* analysis software (Bio-Rad, Hercules, CA, USA). The results were converted into gene copies per mL. All samples were analyzed in experimental (*n* = 3) and technical (*n* = 4 per experimental replicate) replicates.

### 4.4. Data Analysis

The relationship between *GFP* induction and bacteriophage induction (ddPCR) was evaluated using Spearman correlation analysis, considering data across all concentrations and time points after induction. Spearman correlation was chosen because it is a rank-based method that measures the strength and direction of monotonic relationships, making it well-suited for data that may not follow a linear pattern. The scripts used for the analyses were developed in-house using Python3.8.

## 5. Conclusions

Overall, the findings indicate a strong association between the two methods, fluorescence, and ddPCR, at higher concentrations (until 0.25 ng/mL) at all relevant time points (1, 2, and 4 h after induction).

This means that we have developed and validated a GFP-based method, using an *E. coli* model, that can simplify the analysis of phage induction, an important mechanism for AMR spread, which may prove key to furthering our understanding of phages’ role in AMR spread in a One Health setting. This method can be used to screen compounds or environments, such as environmental or clinical samples, chemicals, food, water, etc., opening the door to applications like screening for antimicrobial resistance.

Being a faster, cheaper, efficient, and highly versatile method for researching phage induction adds a layer of sustainability that reinforces the commitment to the One Health approach.

## Figures and Tables

**Table 1:** Primer targets and sequences used for the ddPCR analysis of bacteriophage induction in *E. coli*.

Primer Target	Definition	Sequence
Lambda	Forward	TTGAATGCTGCCCTTCTTCA
	Reverse	CTCTGGCGGTGTTGACATAA
	Probe (FAM)	GCGTCCTGCTGATGTGCTCAGTATCACCGC
16S (EC)	Forward	CATTGACGTTACCCGCAGAA
	Reverse	CGCTTTACGCCCAGTAATTCC
	Probe (HEX)	CGTGCCAGCAGCCGCGGTA

**Figure A2:**
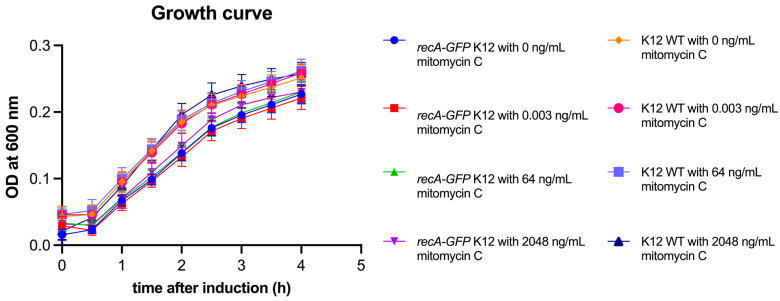
Growth curve for *recA-GFP* K12 *E. coli* and K12 wild-type (WT) *E. coli* in the presence of 0; 0.003; 64; and 2048 ng/mL of mitomycin C.

**Figure A3:**
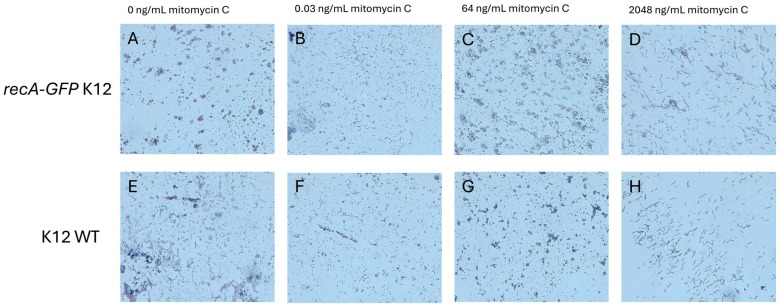
Gram-staining for (**A**–**D**) *recA-GFP* K12 *E. coli* and (**E**–**H**) K12 wild-type (WT) *E. coli* in the presence of (**A**,**E**) 0; (**B**,**F**) 0.003; (**C**,**G**) 64; and (**D**,**H**) 2048 ng/mL of mitomycin C, after 4 h of incubation.

## Data Availability

The data presented in this study are available upon request from the corresponding author. They are not publicly available due to ethical and privacy reasons.

## Abbreviations

The following abbreviations are used in this manuscript:

AMR	Antimicrobial resistance
GFP	Green fluorescent protein
EC	*Escherichia coli*
ddPCR	Droplet digital PCR
WHO	World Health Organization
WT	Wild type
UV	Ultraviolet
ssDNA	Single-strand DNA
MIC	Minimum inhibitory concentration
λ	Lambda phage
PCR	Polymerase chain reaction
LB	Luria–Bertani broth
PBS	Phosphate-buffered saline
OD	Optical density

## Appendix A

Figure A1 Distance correlations. (A) Illustration of the distance correlation coefficients across various mitomycin C concentrations, ranging from 2048 ng/mL to 0.03125 ng/mL, for phage lambda induction and GFP, and (B) distance correlation coefficients for different time points between phage lambda induction and GFP. The data points are plotted to demonstrate the relationship between concentration levels and the strength of correlation. A threshold of 0.7 was established to signify a good correlation when exceeded.

In Figure A2, the growth curve for *recA-GFP* K12 *E. coli* and K12 wild-type (WT) *E. coli* in the presence of 0, 0.003, 64, and 2048 ng/mL of mitomycin C is depicted. The growth curve was obtained by incubating an overnight culture of the respective strains (37 °C, 175 rpm), followed by subculturing (1:20) with fresh LB medium containing varying concentrations of mitomycin C (1:1) (37 °C, shaking before measurements). Measurements were taken every 30 min.

Figure A2 shows similar growth curves within each strain, indicating that mitomycin C does not impact bacterial growth, nor does it differ between strains.

On Figure A3, is possible to see the Gram-staining for *recA-GFP* K12 *E. coli* and K12 wild-type (WT) *E. coli* in the presence of 0, 0.003, 64, and 2048 ng/mL of mitomycin C, after 4 h of incubation. The overnight cultures of the respective strains (37 °C, 175 rpm) were subcultured (1:20) with fresh LB medium containing varying concentrations of mitomycin C (1:1) for 4 h (37 °C, 175 rpm). At the 4 h mark, the Gram-staining protocol described by the manufacturer (Millipore, Merck, Darmstadt, Germany) was followed.

Figure A3 shows similar morphology between strains, indicating that mitomycin C does not impact strain morphology, nor does the presence of the plasmid (*recA-GFP* K12).
